# Mechanisms of Impaired Skeletal Muscle Regeneration and Therapeutic Approaches in Aging and Chronic Disease

**DOI:** 10.3390/ph19071091

**Published:** 2026-07-15

**Authors:** Xia Li, Zihao Zhao, Jiawen Yang, Yijie Zhang, Xinlei Yao, Hualin Sun, Yuntian Shen

**Affiliations:** Jiangsu Key Laboratory of Tissue Engineering and Neuroregeneration, Key Laboratory of Neuroregeneration of Ministry of Education, Co-Innovation Center of Neuroregeneration, Medical School of Nantong University, Nantong University, Nantong 226001, China; 2324310018@stmail.ntu.edu.cn (X.L.); 2331110183@stmail.ntu.edu.cn (Z.Z.); y2934209389@163.com (J.Y.); 2030110218@stmail.ntu.edu.cn (Y.Z.); xinlei_yao@ntu.edu.cn (X.Y.)

**Keywords:** skeletal muscle regeneration, satellite cells, signaling pathways, muscle wasting, aging, therapeutic strategies

## Abstract

Skeletal muscle regeneration is essential for recovery after injury and for maintaining physical and metabolic function. This capacity declines with aging and is often impaired in chronic diseases, limiting effective repair. This review summarizes the main mechanisms that regulate muscle repair, with a focus on satellite cell activity and its interaction with inflammatory, metabolic, vascular, and fibrotic signals at the molecular and cellular level. We discuss how these processes are disrupted in aging, Duchenne muscular dystrophy, chronic obstructive pulmonary disease, diabetes, chronic kidney disease, and cancer cachexia, leading to delayed repair, reduced myogenic differentiation, and fibrosis. We also review current and emerging strategies to improve muscle regeneration, including exercise, bioactive molecules, physical stimulation, gene-based approaches, engineered biomaterials, and cell or cell-derived therapies. Across these conditions, chronic inflammation, metabolic dysfunction, and fibrotic remodeling appear to be common barriers to effective regeneration. Multi-target therapeutic approaches may offer advantages over single-pathway interventions, although clinical evidence is still limited; initial preclinical results, however, are promising.

## 1. Introduction

Skeletal muscle, as a core organ of the motor system and metabolic regulation, maintains the functional muscle fiber groups that account for 40–50% of the body weight of adults through its powerful regenerative capacity [1,2]. This dynamic balance is not only reflected in the adaptive hypertrophy under mechanical stress but also stems from the evolutionarily conserved regenerative program. When mechanical trauma or pathological stress occurs, muscle satellite cells initiate the regenerative program through Pax7-dependent transcriptional activation, and after proliferation, they differentiate into myoblasts to participate in muscle fiber repair [3,4]. The functional plasticity of satellite cells is achieved under the strict regulation of the Wnt/β-catenin and Notch signaling pathways, which play a core role in coordinating the activation and differentiation programs of stem cells. The niche where satellite cells reside, as a highly specialized functional unit, forms a coordinated regulatory network by integrating stem cells, stromal cells, and vascular networks, precisely coordinating the spatiotemporal dynamics of the regenerative process [4,5]. Macrophages regulate the inflammatory response and tissue remodeling stages of the regenerative process through M1-M2 phenotypic conversion [6,7,8]. Fibro-adipogenic progenitors (FAPs) support regeneration through matrix remodeling and paracrine signaling, but abnormally differentiate into fibroblasts to drive fibrosis in chronic inflammation [4,5]. Vascular endothelial cells maintain satellite cells’ activity through the vascular endothelial growth factor A (VEGFA)-FLT1 signal and induce M2 polarization of macrophages through lactate [9]. Pericytes, with both microenvironmental regulatory and myogenic stem cell potential, support the stability of slow muscle fibers [10].

Skeletal muscle regeneration is orchestrated through three interconnected biological phases [11]. The inflammatory phase triggers tissue debridement by neutrophils and M1 macrophages while initiating Pax7-positive satellite cell activation. This cascade transitions into the repair phase, where anti-inflammatory macrophages coordinate MyoD-dependent myoblast proliferation and fusion into multinucleated myotubes, paralleled by provisional extracellular matrix formation and synchronized revascularization through angiogenesis. The process culminates in the remodeling phase with collagen remodeling, myofiber hypertrophy, and neuromuscular junction reconnection, ultimately restoring contractile functionality through integrated tissue adaptation [12,13,14,15]. However, in pathological conditions such as aging, Duchenne muscular dystrophy (DMD), chronic obstructive pulmonary disease (COPD), cancer cachexia, diabetes, and chronic kidney disease (CKD), persistent oxidative stress and chronic inflammatory microenvironments disrupt the regenerative cascade, resulting in critical impairments at multiple regulatory nodes—most notably, reduced self-renewal capacity of muscle progenitor cells, stalled myogenic differentiation, and excessive fibrotic deposition [16,17,18]. Accumulating evidence indicates that these regenerative deficits are strongly associated with diminished physical function, prolonged hospitalization, and increased healthcare burden in affected patients [14]. Unlike the cell-type-centered organization of our group’s recent comprehensive review [7], the present review takes a complementary, pathway-centered approach: rather than organizing around cell types, we structure our discussion around the core signaling pathways governing satellite cell fate and then examine, disease by disease, how these pathways are specifically disrupted across aging, DMD, COPD, diabetes, CKD, and cancer cachexia. Where the literature allows, we distinguish between preclinical and clinical evidence for the therapeutic strategies surveyed in this review, with the aim of providing a clearer picture of translational readiness across this heterogeneous intervention landscape.

Literature search strategy: This narrative review was informed by a literature search of PubMed and Web of Science for articles published from January 2000 to April 2026. Search terms included combinations of: ‘skeletal muscle regeneration,’ ‘satellite cells,’ ‘aging,’ ‘sarcopenia,’ ‘Duchenne muscular dystrophy,’ ‘COPD,’ ‘diabetes,’ ‘chronic kidney disease,’ ‘cancer cachexia,’ ‘signaling pathways’ (Notch, Wnt, TGFβ, mTOR, AMPK), and ‘therapeutic strategies’ (exercise, gene therapy, biomaterials, cell therapy). Reference lists of retrieved articles were hand-searched for additional relevant studies. Priority was given to recent (2018–2026) mechanistic and therapeutic studies, though foundational older references were included where essential for context.

## 2. Signaling Pathways of Muscle Satellite Cell Regeneration

**Figure 1:**
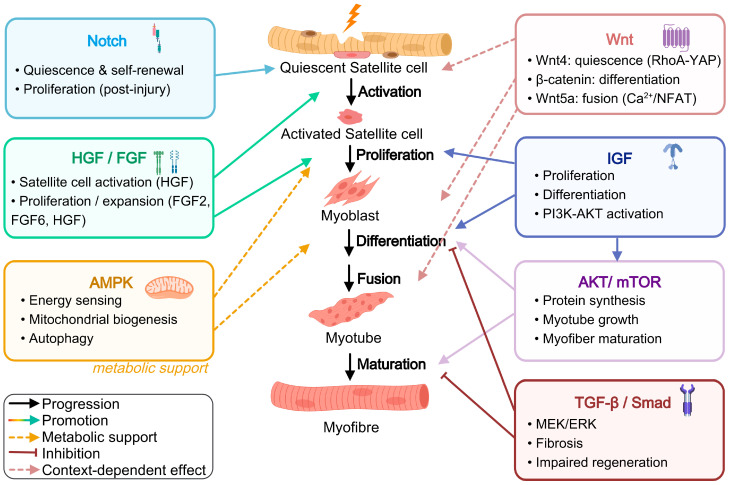
Key signaling pathways regulating satellite cell fate during skeletal muscle regeneration. Following muscle injury, satellite cells progress through activation, proliferation, differentiation, fusion, and maturation to regenerate myofibers. Notch signaling maintains quiescence and self-renewal under homeostatic conditions and supports proliferative expansion after injury. Wnt signaling regulates multiple stages: Wnt4 maintains quiescence via RhoA–YAP, β-catenin drives differentiation, and Wnt5a promotes fusion. HGF and FGF stimulate activation and proliferation; IGF promotes proliferation and differentiation via PI3K–AKT–mTOR; AKT/mTOR supports myofiber growth and maturation. AMPK provides metabolic support through mitochondrial biogenesis and autophagy. TGF-β/Smad signaling inhibits differentiation and promotes fibrosis when dysregulated. Arrows indicate promotion; flat-headed lines indicate inhibition; dashed lines indicate context-dependent effects. Created with Medpeer (http://medpeer.cn, accessed on 1 June 2026).

Satellite cell behavior during muscle regeneration is governed by several interconnected signaling pathways. This section reviews the key pathways involved: Notch–Wnt signaling controls the transition from quiescence to differentiation; AKT/mTOR and AMPK integrate metabolic cues; the TGFβ superfamily regulates the balance between myogenic repair and fibrosis; and insulin-like growth factor (IGF), hepatocyte growth factor (HGF), and fibroblast growth factor (FGF) family members promote progenitor expansion and myofiber maturation (Figure 1).

Notch and Wnt signaling pathways coordinate stage-specific regulation of muscle regeneration [19,20]. The Notch signaling pathway, mediated primarily by receptors Notch1, Notch2, and Notch3, critically regulates skeletal muscle regeneration through precise spatiotemporal control of satellite cell behavior [21,22]. Ligand binding releases the Notch intracellular domain (NICD), which translocates to the nucleus and forms a transcriptional activation complex with RBPJ, inducing downstream target genes Hes1, Hey1, and HeyL that suppress myogenic differentiation factors and sustain satellite cell proliferative competence [20]. Notch1 and Notch2 function synergistically to maintain satellite cell quiescence and niche positioning beneath the basal lamina, primarily by suppressing the myogenic transcription factor MyoD and preventing premature differentiation [21]. Following muscle injury, upregulated Notch ligands, including DLL1 and DLL4 from adjacent myofibers and activated satellite cells, engage Notch1 and Notch2 with increased intensity, driving satellite cell proliferation and expansion [23,24]. Notch1 further sustains muscle stem cell (MuSC) survival during regenerative expansion by activating a Hey1–Mdm2–p53 axis that prevents mitotic catastrophe: Hey1 suppresses Mdm2, stabilizing p53 at levels sufficient to enforce mitotic fidelity without triggering apoptosis [25]. Among these ligands, DLL1 is required for MuSC homeostasis, self-renewal, and regenerative capacity in a cell-intrinsic manner. Beyond its canonical role as a Notch ligand, DLL1 also signals cell-autonomously through its intracellular domain, contributing to both embryonic myogenesis and postnatal muscle repair [26]. In contrast, Notch3 acts as a negative regulator of satellite cell population expansion, potentially through Nrarp-mediated attenuation of Notch1 signaling—a mechanism functionally distinct from the canonical Notch1/2-RBP-J pathway [27]. Within the muscle microenvironment, endothelial-derived DLL4 activates Notch2 in myofibers, inducing atrophy via FoxO signaling [28]. Thus, Notch1 and Notch2 dynamically transition from maintaining quiescence to promoting regenerative expansion in a ligand- and signal strength-dependent manner, while Notch3 constrains proliferation. Their integrated and stage-specific activities are essential for effective muscle repair.

In muscle regeneration, Wnt ligands bind Frizzled receptors and co-receptors, initiating Dishevelled (Dvl)-mediated bifurcation of signals into canonical and non-canonical pathways in sequential phases. The canonical Wnt/β-catenin signaling pathway, mediated by specific ligands such as Wnt3a and Wnt1, drives muscle satellite cell differentiation through dynamic regulation of the GSK3β/β-catenin complex [29]. The pathway directly regulates the myogenic regulatory factor MyoD by facilitating β-catenin/TCF complex binding to its promoter and controls the expression of downstream targets like ENPP2, which promotes myofiber maturation [30,31]. Additional regulatory proteins further stabilize β-catenin activity to facilitate differentiation [32,33]. In non-canonical arm, Wnt7a activates the planar cell polarity (PCP) pathway via Frizzled7 (Fzd7), acting through the Fzd7/Syndecan-4 co-receptor complex and its downstream effector Vangl2 to drive symmetric expansion of satellite stem cells [34]. Wnt5a triggers Ca^2+^/NFAT signaling to enhance myoblast fusion [31,35]. In the maturation phase, Wnt7a/Fzd7 directly activates the Akt/mTOR anabolic pathway through a Gαs/PI3K-containing receptor complex—independently of IGF-receptor activation—inducing myofiber hypertrophy [36,37]. Wnt4 has recently emerged as a critical regulatory hub within the muscle niche, dynamically maintaining satellite cell quiescence via the RhoA-YAP mechanosignaling axis. Its injury-induced downregulation serves as a key permissive signal for initiating the regeneration program [38]. The switch from Notch to Wnt signaling acts as a temporal switch to coordinate muscle regeneration. Notch maintains the proliferation of myogenic progenitor cells, while the activation of Wnt through microenvironment-derived ligands and the enhancement in cellular responsiveness initiate differentiation, ensuring the precision of myogenesis.

As a core pathway for cellular nutrient sensing and growth regulation, the AKT/mTOR signaling pathway plays a dual regulatory role in the metabolic homeostasis and functional regulation of satellite cells. The deletion of Akt1 significantly inhibits the process of muscle hypertrophy by reducing the proliferation level of satellite cells induced by mechanical loading, but does not affect the protein synthesis function mediated by the mTOR pathway [39]. Importantly, mTORC1 integrates nutritional signals such as lysine by phosphorylating downstream molecules like p70S6K, thereby regulating the proliferation, differentiation and autophagy of satellite cells [40,41,42].

Beyond nutrient-sensing machinery, energy stress surveillance is coordinated through complementary metabolic regulators. AMPK acts as a master metabolic regulator of muscle satellite cell function. As an energy sensor, AMPKα1 controls satellite cell activation by adjusting lactate dehydrogenase (LDH) activity to balance energy production and cellular renewal [43,44]. It maintains regenerative capacity through SIRT1/PGC-1α-mediated mitochondrial biogenesis and manages muscle repair quality by coordinating autophagy and fiber-type specialization [45,46,47]. These findings suggest AMPK as a potential therapeutic target in muscle disorders.

The TGFβ superfamily exerts multifaceted regulatory control over muscle regeneration through three primary mechanisms. First, myostatin modulates satellite cell quiescence through Notch–WNT signaling, with genetic depletion altering cell cycle progression [48]. Second, TGFβ inhibits myogenic differentiation through MEK/ERK signaling activation. TGFβ2, WNT9a, and FGFR4 also cross-regulate each other’s expression in satellite cells: TGFβ2 and WNT9a each independently inhibit differentiation, while FGFR4 promotes it. This shifting regulatory balance is thought to underlie the age-related decline in differentiation capacity. TGFβ additionally promotes fibrogenesis via connective tissue growth factor (CTGF)/FGF-2-mediated collagen I synthesis [49,50,51,52]. Third, TGFβ promotes fibrosis through Smad2/3-dependent pathways. The net result of dysregulated TGFβ-family signaling is typically a shift toward fibrosis at the expense of myogenic repair.

IGF further regulates PI3K/AKT/mTOR signaling through damage-responsive splice variants that support proliferation and limit oxidative stress during early regeneration [53]. Dynamic IGFBP6 downregulation early post-injury liberates IGF to override TGFβ-mediated differentiation blockade. Precise pathway modulation is critical—Akt1 deficiency selectively impairs load-induced hypertrophy, whereas mTORC1 over-inhibition disrupts differentiation via cell cycle dysregulation [39,54,55]. IGF signaling links injury-responsive growth cues with AKT/mTOR activity, supporting myoblast proliferation and later myofiber growth. However, excessive mTORC1 activation or premature AKT signaling may impair the proliferation-to-differentiation transition, as shown by mTORC1 over-inhibition disrupting cell cycle progression in vitro.

HGF, a heparin-binding glycoprotein, binds c-Met to activate tyrosine kinase signaling, driving cell proliferation, migration, and tissue repair [53]. In adults, HGF sequesters in muscle Extracellular matrix (ECM) until injury triggers release, activating quiescent satellite cells via paracrine/autocrine mechanisms [56]. HGF-c-Met binding initiates transition from G0 to mTORC1-dependent GAlert state, priming cells for activation [57]. Single-cell RNA sequencing identifies macrophage subset Mac_1 as an HGF source that suppresses Cdkn1b to promote satellite cell proliferation [58]. HGF may therefore prime quiescent satellite cells and support their entry into the regenerative program, although the relative contribution of ECM-derived and macrophage-derived HGF remains unclear.

The FGF family regulates skeletal muscle regeneration through FGFR1/4 receptor signaling [59,60]. FGFR1 triggers ERK/Akt-driven satellite cell proliferation while suppressing differentiation through Myogenin inhibition [59,60]. FGF6 synergizes with FGF2 to expand myoblasts and antagonize fibrosis, effects amplified by HGF co-stimulation [61]. Pathological FGF21 impairs PI3K/Akt signaling, whereas FGF23 promotes myoblast proliferation while repressing terminal differentiation, an effect proposed to expand the proliferative progenitor pool; consistent with this, higher circulating FGF23 is associated with preserved handgrip strength and lower sarcopenia risk in maintenance hemodialysis patients [62,63,64]. Therapeutic approaches focus on modulating FGFR1 activity and calcium signaling pathways, with FGF8b selectively promoting differentiation via ERK activation [62]. Together, these growth factor pathways form an integrated regulatory network that coordinates satellite cell activation, proliferation, and differentiation.

## 3. Impaired Skeletal Muscle Regeneration in Pathological and Aging-Related Conditions

**Figure 2:**
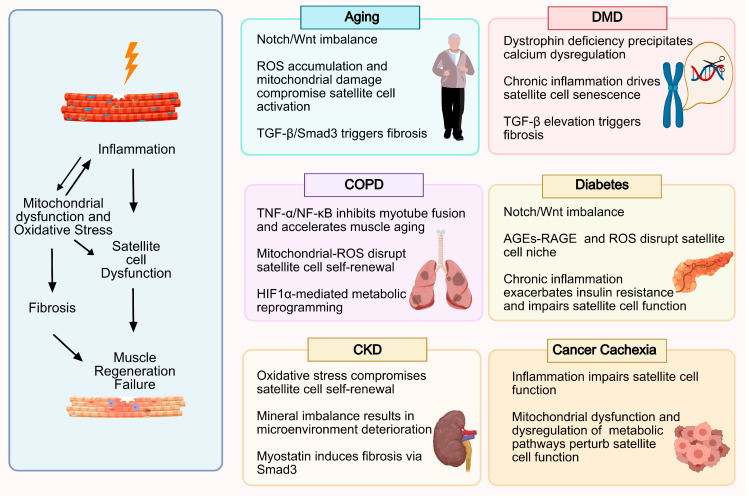
Pathological mechanisms underlying impaired skeletal muscle regeneration in aging and chronic disease. Chronic inflammation, mitochondrial dysfunction, oxidative stress, and progressive fibrosis are shared features that drive regeneration failure across multiple conditions (left panel). In aging, Notch/Wnt imbalance and TGF-β/Smad3-driven fibrosis impair satellite cell activation. In DMD, dystrophin loss causes sarcolemmal instability, chronic inflammation, and satellite cell senescence. In COPD, TNF-α/NF-κB signaling and mitochondrial ROS disrupt satellite cell self-renewal. In diabetes, AGE–RAGE activation and insulin resistance compromise the satellite cell niche. In CKD, oxidative stress and myostatin-mediated fibrosis deteriorate the regenerative microenvironment. In cancer cachexia, tumor-derived cytokines and mitochondrial dysfunction collectively suppress regenerative capacity. DMD, Duchenne muscular dystrophy; COPD, chronic obstructive pulmonary disease; CKD, chronic kidney disease; ROS, reactive oxygen species. Created with Medpeer (http://medpeer.cn, accessed on 1 June 2026).

Skeletal muscle regeneration is impaired in aging and in several chronic diseases, including DMD, COPD, diabetes, CKD, and cancer cachexia. Although these conditions differ in cause and clinical presentation, they share several pathological features that interfere with effective repair, including chronic inflammation, mitochondrial dysfunction, oxidative stress, impaired satellite cell activity, and progressive fibrosis (Figure 2). These changes disrupt myogenic differentiation, matrix remodeling, and vascular support, leading to delayed or incomplete regeneration.

### 3.1. Aging

The regenerative capacity of skeletal muscle declines progressively with advancing age [65]. This decline is characterized by dysregulated activation timing of myogenic transcription factors, a markedly elevated activation threshold of satellite cells, and impaired maturation of newly formed myofibers [66]. Clinically, elderly individuals exhibit prolonged recovery of muscle strength following injury, along with compromised structural integrity and contractile function of regenerated tissue compared to younger counterparts. These deficits are accompanied by fibrotic deposition and lipid infiltration resulting from aberrant extracellular matrix remodeling [67]. The deterioration in regenerative potential is particularly evident in chronic injury models, where the initiation of the repair response is delayed and the regeneration process is prematurely terminated.

Aging-induced impairment in muscle regeneration arises from a complex interplay of multiple contributing mechanisms. First, autonomous dysfunction of muscle satellite cells emerges as a central driver, primarily due to disruption of the Notch–Wnt signaling axis. With aging, there is excessive secretion of Wnt3a, which activates β-catenin signaling to drive myogenic-to-fibrogenic conversion of satellite cells, prematurely suppresses Pax7 expression, and forces exit from quiescence. Concurrently, reduced Fibronectin in the aged niche impairs Wnt7a/Fzd7 signaling, compromising satellite stem cell symmetric expansion. Concurrently, reduced Delta-like1 expression impairs γ-secretase–mediated NICD generation, thereby disrupting Hes1-dependent self-renewal programs [68,69,70]. This signaling imbalance synergizes with abnormal recruitment of DNA methyltransferase DNMT3a, resulting in promoter hypermethylation of key myogenic genes such as Pax7 and transcriptional silencing [71]. Second, metabolic reprogramming fails due to mitochondrial dysfunction. Altered mitochondrial dynamics reduce ATP production efficiency, while excessive reactive oxygen species (ROS) accumulation disrupts proteostasis, inhibiting mTORC1-driven anabolism and impairing autophagic capacity in muscle stem cells [72,73]. NAD^+^ levels decline with age, and restoring NAD^+^ in aged mice reverses this mitochondrial decline [74]. NAD^+^ precursor supplementation rejuvenates aged muscle stem cells and extends lifespan in mice, including in a model of muscular dystrophy [75]. More recently, nicotinamide combined with pyridoxine was shown to stimulate muscle stem cells and accelerate regeneration in aged mice and human myogenic cells [76], with a subsequent randomized controlled trial confirming enhanced muscle stem cell activity and regeneration in humans [77]. Third, microenvironmental regulation becomes dysregulated. Aged FAPs activate Smad3 phosphorylation via TGF-β1, promoting fibrosis and suppressing MuSC proliferation [78,79,80]. FAPs also engage macrophages through complement factor C3: FAP-secreted C3 supports monocyte/macrophage survival, metabolic activity, and phagocytic clearance of necrotic myofibers, and plasma C3 levels are reduced in sarcopenic older adults, suggesting this axis may decline with age [81]. Notably, immune-regulatory pathways also deteriorate during aging. Midbrain astrocyte-derived neurotrophic factor (MANF)-mediated immunomodulation is compromised, and its absence after injury correlates with regeneration failure [82]. Combined with elevated 15-hydroxyprostaglandin dehydrogenase (15-PGDH) activity that depletes Prostaglandin E2 (PGE2), this creates a pro-fibrotic niche [83]. Together, these changes impair satellite cell activation, alter metabolism, and promote fibrosis in aged muscle.

Emerging pharmacological strategies targeting cellular senescence offer promising avenues for rejuvenating the aged muscle niche. The progressive accumulation of senescent cells, including dysfunctional FAPs, exhausted satellite cells, and inflammatory macrophages, drives the secretion of the senescence-associated secretory phenotype (SASP), which further suppresses satellite cell function and amplifies fibrotic signaling [84]. Senolytic agents, such as the BCL-2 family inhibitor navitoclax (ABT-263) and the dasatinib/quercetin combination, selectively eliminate senescent cells from the muscle interstitium, thereby reducing SASP-mediated inflammation and restoring regenerative capacity [80]. In preclinical aging models, periodic senolytic treatment has been shown to improve muscle stem cell activation kinetics, enhance myofiber regeneration, and extend healthspan [80]. Furthermore, the senomorphic compound rapamycin, by inhibiting mTORC1, delays the onset of satellite cell senescence and maintains their quiescent state, thereby preserving the long-term regenerative reservoir [72]. Notably, senescence-related regenerative decline recurs across multiple pathological contexts discussed in this review, including hypoxia-driven senescence in COPD (Section 3.3) and exercise-modulated FAP senescence (Section 4.1), underscoring cellular senescence as a shared node for therapeutic intervention across diverse muscle pathologies. These findings support further evaluation of senotherapeutic approaches for age-associated regenerative failure, particularly in combination with exercise or PGE2-related interventions.

### 3.2. Duchenne Muscular Dystrophy (DMD)

DMD is a severe X-linked genetic disorder characterized by progressive degeneration of skeletal muscle fibers and defective regeneration [85,86]. The loss of functional dystrophin compromises sarcolemmal stability, rendering muscle fibers highly susceptible to mechanical damage during contraction [87].

Regeneration failure in DMD results from a combination of intrinsic stem cell defects and extrinsic pathological influences including chronic inflammation and fibrosis. Muscle stem cells in DMD exhibit fundamental functional impairments, including disrupted polarity and diminished stemness, which compromise their differentiation capacity [16]. These cells display early-onset senescence phenotypes that further restrict their regenerative potential [88]. Recent studies using pig DMD models have revealed enhanced traction forces in satellite cells [89], while single-cell RNA sequencing of mdx/D2-mdx mice uncovered gene expression dysregulation leading to differentiation arrest and imbalances in autophagy and cellular aging. Targeting autophagy pathways has been proposed as a promising strategy to restore myogenic function [86]. Chronic inflammation exerts dual effects in DMD [87]. While moderate inflammation aids in clearing necrotic debris and initiating repair, persistent inflammation exacerbates tissue damage and suppresses regeneration. Neutrophils contribute to prolonged inflammation through cytokine release, worsening disease progression. Fibrosis is another major barrier to effective regeneration. Accumulation of FAPs and overactivation of the TGF-β signaling pathway drive excessive extracellular matrix deposition, creating a restrictive microenvironment for muscle repair [85]. Anti-inflammatory and anti-fibrotic strategies may complement dystrophin-restoring therapies, although their ability to produce durable functional improvement remains uncertain.

### 3.3. Chronic Obstructive Pulmonary Disease (COPD)

Patients with COPD and concomitant sarcopenia exhibit exacerbated clinical manifestations, including increased dyspnea index, reduced muscle strength, and diminished exercise capacity, all strongly correlated with elevated all-cause mortality [90]. Skeletal muscle dysfunction in COPD is characterized by two major pathological features: fiber-type remodeling and disrupted regenerative compensation. Specifically, oxidative slow-twitch fibers are progressively replaced by glycolytic fast-twitch fibers, accompanied by microcirculatory degeneration, contributing directly to muscle wasting and functional decline [91,92]. Despite evidence of compensatory regenerative activation, such as increased central-nucleated fibers and upregulated MyoD expression, the terminal differentiation regulator Myogenin is suppressed, leading to defective myofiber maturation [93]. Satellite cell dysfunction is central to this pathology: chronic inflammation and hypoxia accelerate cellular senescence and induce molecular imbalances during proliferation and differentiation stages [94]. Although activation signals drive entry into the proliferative phase, autophagy defects and mitochondrial dysfunction ultimately impair terminal differentiation, resulting in a paradoxical state of overactivation without effective regeneration.

The core pathological network in COPD-associated muscle dysfunction is driven by inflammatory mediators and mitochondrial abnormalities [95,96,97]. TNF-α activates macrophage NF-κB signaling, promoting M1 polarization that inhibits myotube fusion and accelerates muscle aging. Concurrently, hypoxia triggers glycolytic reprogramming via the HIF1α/VEGF axis, which, together with Histone Deacetylase 2 (HDAC2) inhibition, establishes a self-amplifying NF-κB loop. This results in a distinct phenotype featuring high HIF1α/VEGF and low HDAC2 expression, closely mirroring clinical observations [98,99]. Mitochondrial dysfunction serves as a critical hub in this process. Accumulation of succinate and 2-hydroxyglutarate inhibits α-ketoglutarate-dependent dioxygenases, leading to DNA hypermethylation and HIF signaling disruption [100]. Simultaneously, excessive ROS activates the FOXO-MuRF-1/atrogin1 axis and perturbs Notch signaling, impairing satellite cell renewal [94,101]. Collectively, these converging defects in mitochondrial quality control, redox balance, and epigenetic regulation compound the progressive loss of regenerative capacity characteristic of COPD-associated muscle wasting.

### 3.4. Diabetes

Diabetes mellitus is a chronic metabolic disorder characterized by dysregulated glucose homeostasis, classified into type 1 (absolute insulin deficiency) and type 2 (insulin resistance) [102]. Hyperglycemia impairs muscle regeneration through multiple mechanisms: it induces structural alterations in skeletal muscle, including myofiber atrophy, mitochondrial dysfunction, and reduced oxidase activity; promotes fiber-type switching; and delays post-injury repair, manifesting as delayed maturation of regenerating fibers and excessive extracellular matrix fibrosis [103,104,105]. Collectively, these changes lead to diminished muscle strength and exercise tolerance [106,107,108,109], culminating in a multi-level breakdown of the muscle regeneration compensatory mechanism.

Diabetic muscle regeneration impairment arises from pathological cascades involving toxic microenvironment, metabolic dysregulation, and disrupted signaling networks. Advanced glycation end-products accumulate (AGEs) in skeletal muscle, binding to Receptor for Advanced Glycation End-products (RAGE) receptors to chronically activate pro-inflammatory pathways while physically altering the satellite cell niche [110,111]. Concurrently, cytokines including IL-6 and TNF-α exacerbate insulin resistance by disrupting IRS-1 phosphorylation and directly suppressing satellite cell functionality [107,112,113,114,115]. ROS-induced mitochondrial collapse and hyperglycemia-driven pericyte loss synergistically impair tissue regeneration through apoptotic activation and compromised vascular nutrient supply [116,117,118]. Compounding these insults, diabetes suppresses endogenous protective mechanisms including the RISK and SAFE pathways while reshaping bone marrow microenvironments through vascular rarefaction and adipose infiltration, further depleting regenerative progenitor pools [115,119]. In diabetes, Wnt/β-catenin signaling is downregulated, particularly its β-catenin and TCF4 components. Notch signaling, meanwhile, fails to follow its normal cycle of activation and downregulation, instead staying persistently active. This sustained, dysregulated activity is unlike the transient activation that supports self-renewal in healthy muscle. It impairs satellite cell function and delays myogenic differentiation [120,121,122]. These interlinked mechanisms encompassing chronic inflammation, metabolic stress, microvascular collapse, and stem cell fate reprogramming establish an irreversible molecular switch driving progressive muscle atrophy and regeneration failure in diabetes.

### 3.5. Chronic Kidney Disease (CKD)

CKD-associated skeletal muscle pathology is primarily characterized by impaired regeneration linked to metabolic network imbalance. Mitochondrial complex I dysfunction in CKD leads to excessive ROS production and widespread activation of oxidative stress markers (e.g., 8-OHdG, Nox4, SDH) [123,124,125]. This hostile microenvironment directly compromises satellite cell self-renewal, evidenced by reduced Pax7 expression and elevated levels of differentiation markers MyoD and Myogenin, indicating blocked initiation of the regeneration program [123]. Disrupted calcium-phosphorus homeostasis via the parathyroid hormone axis further impairs skeletal muscle-bone crosstalk, weakening tissue repair capacity [126]. Myostatin induces FAP fibrotic transformation via Smad3 signaling, contributing to skeletal muscle fibrosis and impaired regenerative capacity [127]. Enhanced protein degradation through the ubiquitin-proteasome system and myostatin-mediated catabolism, coupled with substrate depletion from protein-energy wasting, creates a catabolic-anabolic vicious cycle [128,129,130,131]. Collectively, mitochondrial oxidative stress, mineral imbalance, and proteolytic activation result in satellite cell dysfunction and microenvironment deterioration, culminating in irreversible regenerative decompensation in CKD.

### 3.6. Cancer Cachexia

Cancer cachexia is a multifactorial syndrome marked by progressive weight loss and profound skeletal muscle atrophy. It significantly diminishes patient quality of life and often limits the efficacy of anti-cancer therapies. Emerging evidence highlights that cancer-associated muscle wasting involves not only accelerated catabolism but also impaired regenerative capacity, primarily driven by satellite cell dysfunction and metabolic dysregulation [2,132].

The tumor microenvironment releases pro-inflammatory cytokines (e.g., TNF-α, IL-6, IL-1β) and metabolic toxins that perturb satellite cell function. These include abnormal Pax7 accumulation, MyoD-mediated epigenetic silencing, and dysregulation of key metabolic pathways such as AMPK/SIRT1 and IGF-1/mTOR [133,134,135,136]. Mitochondrial dysfunction further exacerbates energy deficiency, impairing the bioenergetic support required for effective regeneration [137]. Concurrently, immune microenvironment alterations occur: the transition from M1 to M2 macrophage polarization is impaired, while neutrophil infiltration persists, both of which amplify regenerative failure [138,139]. Recent findings indicate that Epsti1 regulates IFN-γ-JAK-STAT1 signaling by mediating VCP-dependent STAT1 degradation, thereby mitigating inflammation and promoting muscle regeneration in cancer cachexia [140]. In pancreatic cancer-associated cachexia models, the accumulation of senescent cells correlates strongly with regenerative dysfunction. Temporal upregulation of CDKN1A/p21 reflects accelerated aging and contributes to immune microenvironment disruption, ultimately inhibiting satellite cell activation [141]. These insights underscore the importance of enhancing muscle regenerative capacity as a therapeutic strategy in cancer cachexia.

## 4. Skeletal Muscle Regeneration Strategies

**Figure 3:**
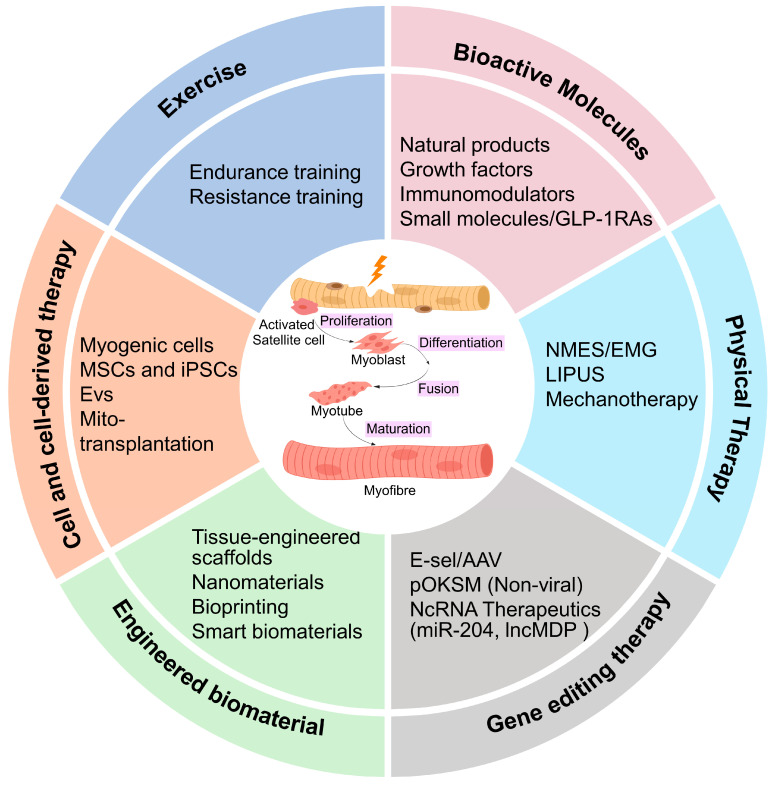
Current and emerging therapeutic strategies for skeletal muscle regeneration. Six major intervention categories are shown. Exercise (endurance and resistance training) enhances satellite cell activity and delays senescence. Bioactive molecules include natural products, growth factors, immunomodulators, and small molecules such as GLP-1 receptor agonists. Physical therapy modalities, including NMES/EMG stimulation, LIPUS, and mechanotherapy, promote myogenic activity through mechanical and electrical cues. Gene-editing strategies encompass viral (E-sel/AAV), non-viral (pOKSM), and ncRNA-based approaches (miR-204, lncMDP). Engineered biomaterials range from scaffolds and nanomaterials to smart biomaterials with programmable immunomodulatory properties. Cell and cell-derived therapies include myogenic cells, MSCs, iPSCs, EVs, and mitochondrial transplantation. The central diagram illustrates the satellite cell regenerative cascade from activation through myofiber maturation. EVs, extracellular vesicles; LIPUS, low-intensity pulsed ultrasound; NMES, neuromuscular electrical stimulation; MSCs, mesenchymal stem cells; iPSCs, induced pluripotent stem cells. Created with Medpeer (http://medpeer.cn, accessed on 1 June 2026).

Skeletal muscle regeneration serves as a pivotal repair mechanism in clinical rehabilitation, encompassing diverse therapeutic strategies such as exercise-based rehabilitation programs, bioactive molecule regulation, physical therapy modalities, gene-editing technologies, engineered biomaterials, and cell and cell-derived therapies (Figure 3, Table 1). These interventions synergistically enhance musculoskeletal function by promoting myofiber remodeling, neurovascular integration, and extracellular matrix reorganization. This review systematically examines the mechanistic basis and translational potential of each regenerative strategy, while critically evaluating their clinical feasibility and existing challenges.

### 4.1. Exercise

Exercise remains the most widely utilized intervention for enhancing skeletal muscle regeneration, exerting multi-dimensional physiological effects across hemodynamic, cellular, and molecular levels [165]. Mechanistically, repetitive mechanical loading increases capillary density and oxygen delivery to injured tissues, thereby improving nutrient perfusion and metabolic support. At the stem cell level, Piezo1-mediated mechanotransduction facilitates directional migration of satellite cells. Resistance training enhances protein synthesis via mTORC1 activation [142,143,144,166], whereas endurance training delays satellite cell senescence through modulation of miR-1 and miR-31 expression [142,144]. On the microenvironmental level, sustained AMPK activation during exercise preserves mitochondrial homeostasis via the PINK1-Parkin pathway [167] and synergizes with pharmacological agents to reverse FAP apoptosis resistance, thereby enhancing regeneration in chronic myopathy models [168].

Exercise induces FAP senescence, which suppresses NLRP3 inflammasome activation and CCN2-driven fibrosis [107,169,170], establishing a dual immunometabolic regulatory network. Endurance exercise further activates p38 MAPK signaling to upregulate TAZ expression, which collaborates with Pax7 to induce Myf5 transcription and activate the mTOR pathway, thereby promoting satellite cell activation and myofiber regeneration [171]. Long-term exercise interventions confer broad-spectrum benefits by integrating these multi-target mechanisms, significantly improving muscle metabolism and preventing disuse atrophy—offering a safe, accessible, and universally applicable strategy for clinical rehabilitation.

### 4.2. Bioactive Molecules

Pharmacological interventions represent a crucial pillar in muscle regeneration therapy. Natural compounds show promise: Asperosaponin VI from Dipsacus root mitigates interstitial fibrosis and promotes fiber remodeling via GSK-3β inhibition, while Andrographolide lactone enhances myotube differentiation through histone H3K4me3 modification [172,173]. Chestnut flower extract containing ellagic acid and polyamines improves autophagy by activating AMPK and suppressing Atg5 acetylation, thereby optimizing the regenerative niche [174]. Ginkgolide B restores bone-muscle endocrine crosstalk via osteocalcin-GPRC6A signaling, offering structural support by inhibiting fibrotic remodeling [175]. Traditional Chinese medicine formulations such as Bu Yang Tang activate the CXCR4-GPX4 axis to inhibit ferroptosis and enhance satellite cell proliferation and differentiation, effectively improving muscle strength in ovariectomy-induced sarcopenia models [176,177]. Non-steroidal anti-inflammatory drugs (NSAIDs) such as celecoxib have demonstrated protective effects against both denervation-induced and hindlimb unloading-induced muscle atrophy by suppressing inflammation, oxidative stress, and improving microcirculation [178,179].

Growth factors play central roles in myogenesis: IGF-1 outperforms IGF-2 in stimulating myoblast proliferation and protein synthesis via PI3K/Akt/mTOR activation [180,181,182,183]. FGF family members regulate myogenic precursor fate through MAPK/ERK signaling, while HGF initiates satellite cell activation via c-Met and integrin-FAK pathways, concurrently modulating macrophage polarization to improve inflammation [58,184,185]. A newly identified myokine, FNDC1, binds integrin α5β1 to activate FAK/PI3K/AKT/mTOR signaling, directly promoting myoblast differentiation and myotube fusion [186]. Resolvins exhibit distinct regenerative functions: Resolvin D1 polarizes macrophages toward a pro-regenerative phenotype, indirectly enhancing tissue repair, whereas Resolvin D2 directly regulates fiber type specification, promoting dominant fiber reconstruction at injury sites [149,150]. PGE2 targets EP4 to activate cAMP response element-binding protein (CREB) phosphorylation and inhibit 15-PGDH, reversing epigenetic dysregulation in aged satellite cells through three key mechanisms: first by restoring juvenile transcriptional networks and mitochondrial bioenergetics; second by establishing a molecular memory that preserves regenerative capacity within aging microenvironments; and third by demonstrating rapid efficacy in enhancing muscle mass and strength in elderly subjects, particularly when synergized with eccentric exercise regimens [83,145]. Forskolin promotes satellite cell proliferation and reduces fibrosis in dystrophic mice but is limited by cardiac toxicity upon prolonged use [187]. The heparan sulfate mimetic RGTA^®^ accelerates post-injury muscle regeneration by increasing myonuclear content and vascularization in regenerating fibers. In vitro studies demonstrate its direct effects on both satellite cells and endothelial cells, promoting myotube fusion and angiogenesis, respectively [188]. Melatonin combined with exercise rescues Bmal1 knockout-induced regeneration deficits, highlighting its role in preserving circadian-regulated muscle integrity and mitochondrial function [189]. Emerging evidence highlights sarcosine’s role in age-related regeneration: it activates GCN2 signaling to drive anti-inflammatory macrophage polarization, simultaneously enhancing energy metabolism and insulin sensitivity [190]. This dual immunometabolic effect provides a novel framework for developing sarcosine-based interventions targeting sarcopenia in the elderly. GCN2 functions as an amino acid sensor that becomes activated when intracellular amino acid pools are depleted. In models of denervation-induced atrophy, GCN2 activation drives FoxO3a-mediated muscle wasting, and correcting amino acid deficiency, for example through essential or branched-chain amino acid (BCAA) supplementation, protects against this form of atrophy [191]. In age-related sarcopenia, however, the picture is more complex. Multi-omic profiling has identified impaired mitochondrial BCAA catabolism, rather than simple BCAA deficiency, as a causal driver of sarcopenia. Pathological BCAA accumulation overactivates mTOR and impairs autophagy and mitochondrial function, and enhancing BCAA catabolism, rather than further BCAA supplementation, improved muscle mass and strength in aged mice [192]. These findings, highlighted as ‘an mTOR paradox in sarcopenia’ by an accompanying commentary [193], suggest that nutritional strategies for preserving muscle mass may need to be tailored to the underlying cause of amino acid dysregulation rather than assuming a uniform deficiency state.

Of particular clinical relevance is the growing intersection between anti-obesity pharmacotherapy and skeletal muscle regeneration. Glucagon-like peptide-1 receptor agonists (GLP-1RAs), including semaglutide and liraglutide, are now first-line therapies for obesity and type 2 diabetes, yet their impact on muscle tissue presents a double-edged challenge. On one hand, GLP-1RAs improve insulin sensitivity, reduce ectopic lipid infiltration in skeletal muscle, and attenuate systemic inflammation—all of which can improve the myogenic microenvironment [194]. On the other hand, recent large-scale clinical evidence indicates that approximately 25–39% of GLP-1RA-induced weight loss constitutes lean mass rather than adipose tissue [147]. Using a high-fat diet mouse model, Nalbandian et al. showed that semaglutide treatment impairs satellite cell function and post-injury muscle strength recovery, a deficit linked to suppressed PGE2 signaling; co-administration of a 15-PGDH inhibitor, which elevates endogenous PGE2 levels, restored satellite cell-mediated regeneration and strength recovery in semaglutide-treated mice [146]. This preclinical rationale has since translated to the clinic: the Phase 2 COURAGE trial (NCT06299098) is evaluating semaglutide combined with the anti-myostatin antibody trevogrumab, with or without the anti-activin A antibody garetosmab, and interim results indicate that combination therapy substantially preserves lean mass while enhancing fat loss relative to semaglutide alone [147,148]. These findings suggest that muscle-protective strategies may be needed alongside anti-obesity treatments, although clinical evidence remains limited. Targeted delivery systems, including ligand-directed nanoparticles or engineered exosomes, may help reduce off-target effects, but these approaches remain largely preclinical.

### 4.3. Physical Therapy

Electrical stimulation has emerged as a promising non-invasive modality for muscle regeneration. Innovative Electromyography (EMG)-derived “noise” stimulation (EMGstim) promotes muscle progenitor differentiation via endogenous ATP release, showing superior efficacy in traumatic or neuropathic injuries compared to conventional protocols [151]. Neuromuscular electrical stimulation (NMES) enhances myogenic precursor proliferation and fusion in elderly subjects, improving regeneration outcomes while reducing oxidative stress and enhancing functional recovery [152,195]. Distal electrical stimulation accelerates neuromuscular junction preservation and satellite cell differentiation. Single-cell RNA sequencing reveals upregulated genes related to myogenesis and angiogenesis in stimulated satellite cell clusters, indicating a favorable regenerative microenvironment [196]. Low-frequency stimulation early after muscle strain in rats enhances MyoD and myogenin expression, accelerating structural restoration [197]. Together, these findings underscore the multifaceted benefits of electrical stimulation, warranting further optimization of stimulation parameters for enhanced clinical translation.

Low-intensity pulsed ultrasound (LIPUS) exerts multi-level effects on muscle regeneration: it activates AMPK to regulate satellite cell and myoblast activity, stimulates Wnt/β-catenin signaling to promote M2 macrophage polarization and improve local immunity [198]. LIPUS also modulates ECM remodeling by increasing the TIMP-1/MMP-3 ratio [153,199]. Through this triad of actions—”direct cellular stimulation, molecular pathway modulation, and microenvironment optimization”—LIPUS offers a robust platform for multi-targeted muscle regeneration. Future research should focus on parameter refinement and combination therapies to maximize its clinical impact.

A promising frontier in physical therapy is robotic mechanotherapy. Recent work by Seo et al. demonstrated that precisely controlled cyclic compressive loading delivered via a soft-interface robotic device dramatically improved functional recovery of severely injured skeletal muscle in mice. The mechanism involves rapid clearance of neutrophil populations and their associated pro-inflammatory mediators from the injury site, thereby accelerating the transition to the myogenic repair phase. This robotic approach, compatible with real-time ultrasound imaging for tissue strain monitoring, provides a reproducible and quantifiable mechanotherapy protocol that bridges the gap between empirical physical therapy and mechanistically informed rehabilitation [154]. Complementing this, computational agent-based models of muscle regeneration have now been validated to simulate injury-type-specific cellular and cytokine dynamics across cardiotoxin, freeze-induced, and eccentric contraction injury models, revealing that each injury triggers unique microenvironmental interactions that influence regeneration particularly around 28 days post-injury [200]. Such computational frameworks are beginning to guide the rational design of targeted therapeutic strategies in preclinical studies, representing a new interface between system biology and physical rehabilitation medicine.

### 4.4. Gene-Editing Therapy

Gene therapy strategies for skeletal muscle regeneration are overcoming the limitations of conventional treatments through innovative targeting approaches and technological advancements. E-selectin gene therapy delivered via adeno-associated virus vectors (E-sel/AAV) not only enhances vascular reconstruction in ischemic limbs but also directly stimulates the proliferation and differentiation of muscle progenitor cells, leading to a significant increase in slow-twitch muscle fiber content and improved motor function recovery [155]. Non-viral methods for delivering cell reprogramming technologies offer novel avenues for skeletal muscle regeneration. Local injection of plasmid DNA encoding OKSM (pOKSM) transcription factors into mouse skeletal muscle has successfully induced transient pluripotent-like cellular states and enhanced proliferation. This approach accelerates muscle regeneration while reducing fibrosis, highlighting its potential for tissue repair [201]. The regulation of non-coding RNAs (ncRNAs) provides new insights into regenerative medicine. For example, inhibition of miR-204 promotes myoblast proliferation, migration, and differentiation by alleviating its suppressive effects on key regeneration-related factors such as Pax7 and IGF1; overexpression of miR-24-3p facilitates myotube fusion and injury repair by downregulating myogenesis-inhibitory genes like Nek4 and Pim1 [156,202]; lncMDP1 enhances the expression of cystine/glutamate transporter CHAC1 via a molecular sponge mechanism, forming a positive feedback loop that drives muscle regeneration; conversely, lncMPD2 suppresses myoblast cell cycle progression through the miR-34a-5p/THBS1 axis, and its genetic deletion significantly enhances myoblast proliferation and differentiation, thereby promoting muscle regeneration [157,203]. Targeting core molecular pathways involved in regeneration, studies have shown that suppression of the DBC1-FOXO3 axis mitigates muscle atrophy, activation of CLIC5 promotes myogenic differentiation via modulation of the Wnt/β-catenin pathway, and upregulation of ZEB1 not only facilitates macrophage polarization toward an anti-inflammatory phenotype but also supports the quiescence and regenerative capacity of muscle stem cells [204,205,206]. Additionally, CREG1 has been identified as a critical regulator that maintains mitochondrial autophagy homeostasis to support energy metabolism during muscle regeneration, acting through the AMPKα1-C-CBL signaling axis to promote satellite cell differentiation and new myofiber formation; targeted interventions targeting this pathway can effectively reverse regenerative deficits, offering a novel therapeutic strategy for muscle degenerative disorders [207,208]. These findings elucidate the regulatory mechanisms underlying skeletal muscle regeneration and provide a theoretical foundation for the development of precise gene-editing tools. Modulating these targets enables optimization of the regeneration process across multiple dimensions, including metabolic regulation, differentiation induction, and microenvironmental remodeling.

### 4.5. Engineered Biomaterials

Tissue engineering has significantly advanced skeletal muscle regeneration through the synergistic design of multi-dimensional biomaterial systems [13,14]. The release of bioactive silicon ions from 80Si-BGN effectively promotes myogenic differentiation of myoblasts, highlighting its promising role in regulating tissue regeneration [159]. Decellularized extracellular matrix (dECM)-derived hydrogels closely mimic the native extracellular matrix microenvironment, thereby enhancing cell adhesion, proliferation, differentiation, and tissue morphogenesis—offering novel strategies for skeletal muscle repair [209]. In the fabrication of dECM-based materials, the combination of pH-responsive fiber self-assembly with shear flow-controlled alignment technology enables precise structural construction, resulting in the successful development of oriented scaffolds with biomimetic nano-topographical features that significantly improve mechanical coupling and functional integration between muscle fibers and the surrounding matrix [210].

The application of nanomaterials has uncovered unique regulatory mechanisms in skeletal muscle regeneration [211,212]. For example, gold nanoparticles enhance the regenerative microenvironment by inducing macrophage polarization toward the anti-inflammatory M2 phenotype [158]; degradable polycitrate nanoclusters (POCG-PEI600) promote myoblast proliferation and myotube formation via specific activation of the p38 MAPK pathway, while maintaining excellent biocompatibility and safety [213]. Furthermore, polysaccharide-based double-crosslinked cryogels (FuMA/GelMA), characterized by their hierarchical porous architecture, not only support muscle fiber regeneration but also reduce collagen deposition and accelerate neovascularization [214]. Electrospun nanofibers provide effective physical cues for myoblast adhesion, proliferation, and differentiation by accurately replicating the anisotropic topological structure of the natural extracellular matrix [215]. Sandwich-like electrospun nanofiber scaffolds further enhance regenerative potential by guiding cellular mechanical remodeling through aligned outer fibers and modulating macrophage polarization via hyaluronic acid-mediated signaling in the inner layer, thereby constructing a favorable immunomodulatory microenvironment [216].

The integration of 3D bioprinting and nanomaterial technologies offers cross-scale solutions for reconstructing complex tissue architectures. Biodegradable polyurethane urea elastomers fabricated via 3D printing can recapitulate the structural and functional properties of skeletal muscle, promoting the formation of muscle-like tissue in rat tibialis anterior defect models and significantly enhancing regeneration outcomes [217]. Artificial muscle constructs generated using microvalve-assisted coaxial 3D bioprinting, comprising C2C12 myoblast aggregates encapsulated within a photocrosslinkable hydrogel shell, mimic the anatomical and physiological characteristics of native skeletal muscle. Upon implantation into immunodeficient rat models, these constructs exhibited robust responses to electrical stimulation and achieved histologically confirmed tissue regeneration [218]. GHM bioink, developed by incorporating MXene nanoparticles into a GelMA/HAMA composite hydrogel, demonstrates superior printability, cytocompatibility, and microstructural fidelity. This formulation significantly enhances the skeletal muscle differentiation of C2C12 cells and exhibits enhanced regenerative performance with minimal immune rejection in volumetric muscle loss models [160]. Collectively, the integration of tissue engineering scaffolds, functional nanomaterials, and 3D bioprinting has enabled the establishment of multi-tiered regenerative medicine strategies. These diverse material systems work synergistically to regulate cellular behavior, optimize biomechanical environments, and coordinate biochemical signaling, offering structurally adaptable and functionally responsive solutions for clinical translation.

Emerging in 2026, programmable biomaterials with spatiotemporally controlled immunomodulatory properties represent the next generation of scaffold design for musculoskeletal regeneration. Liang et al. demonstrated that spatiotemporal delivery of immunomodulatory signals via programmable biomaterials sequentially suppresses the early pro-inflammatory phase and then promotes the anti-inflammatory, pro-regenerative transition, mimicking the natural immune cascade with a degree of precision not previously achievable with static scaffolds [161]. Concurrently, a 3D printing strategy employing cyclic strain-driven engineering with enhanced viscoelastic ECM bioink enables the fabrication of skeletal muscle blocks whose mechanical and biological properties closely recapitulate native muscle, showing robust in vivo myogenesis in volumetric muscle loss models [161]. These advances highlight a shift from passive structural scaffolds toward active, stimulus-responsive biomaterial systems capable of dynamically instructing the regenerative niche at multiple stages of repair.

### 4.6. Cell and Cell-Derived Therapies

Current therapeutic approaches for skeletal muscle injury predominantly employ myogenic and non-myogenic cell types [219]. Myogenic cells, such as satellite cells, possess the direct capacity to differentiate into new myofibers. Their key advantages include the ability to initiate regeneration at low cell doses and to sustain the stem cell pool through self-renewal mechanisms. Muscle-derived stem cells (MDSCs) and pericytes exhibit both multi-lineage differentiation potential and paracrine signaling functions, making them particularly effective for repairing complex tissue injuries. Notably, satellite cells display functional heterogeneity [220], with recent studies identifying a Gli1+ subpopulation that demonstrates enhanced mTOR signaling activity, increased mitochondrial content, and a distinct “GAlert” rapid activation phenotype—features that collectively contribute to superior regenerative performance [221]. Non-myogenic strategies primarily involve mesenchymal stem cells (MSCs) and induced pluripotent stem cells (iPSCs). MSCs exert their therapeutic effects through immunomodulation and secretion of trophic factors that improve the local microenvironment, while iPSCs enable scalable production of autologous myogenic progenitors via reprogramming techniques, thereby circumventing donor cell limitations [222,223]. Despite these advances, clinical translation remains constrained by challenges including limited cell accessibility, phenotypic drift during in vitro expansion, and a lack of consensus on dosing protocols.

Emerging strategies aim to enhance cellular efficacy and refine delivery methodologies [224]. Engineering biomaterial-based microenvironments and modulating cell–matrix interactions have been shown to significantly improve mesenchymal stem cell survival rates and paracrine activity while also reshaping their secretory profiles [225,226,227,228]. For example, extracellular vesicles (EVs) and soluble protein/peptide factors derived from human gingiva-derived mesenchymal stem cells (iGMSCs) under three-dimensional optimized culture conditions are markedly enriched. These bioactive components demonstrate differential expression patterns in pathways associated with oxidative phosphorylation, Wnt/β-catenin signaling, Notch signaling, and inflammatory regulation [162], which together augment myogenic induction and confer enhanced anti-inflammatory effects on macrophages. Compared to conventional cell transplantation, EVs preserve the therapeutic benefits of their parent cells while minimizing immune rejection risks, positioning them as promising tools in muscle regeneration research [222]. Recent advancements include the development of a biomimetic delivery platform integrating hiPSC-derived myofiber exosomes with a tyramine-gelatin hybrid hydrogel (GelTA). This system employs in situ crosslinking to achieve sustained EV release and dynamic modulation of the extracellular matrix microenvironment, resulting in robust upregulation of myogenic markers and functional reconstruction of damaged muscle tissue in preclinical models [229]. Furthermore, EVs engineered to enhance N-cadherin-mediated interactions significantly improve endothelial and myoblast viability, migration, and morphogenesis. Encapsulation of such exosomes within ischemia-targeted platelet membranes followed by intravenous administration has demonstrated restoration of hindlimb perfusion in murine models within 28 days, accompanied by marked enhancement in skeletal muscle regeneration [230].

Skeletal muscle regeneration critically depends on mitochondrial energy metabolism and homeostasis, with functional deficits in mitochondria exacerbating post-injury degeneration [231]. Emerging evidence demonstrates that exogenous mitochondrial transplantation effectively addresses these impairments across diverse injury models. In rodent rotator cuff injuries, intramuscular delivery of BMSC-derived mitochondria (BMSC-Mito) or muscle-derived mitochondria restored muscle mass by enhancing oxidative phosphorylation and attenuated fibrosis through antioxidant activation [163,232]. Local mitochondrial transplantation in sciatic nerve crush models effectively mitigated denervation-induced apoptosis and oxidative stress, significantly enhancing muscle mass recovery and the functional restoration of neuromuscular junctions and motor units [233]. Systemic mitochondrial administration accelerated functional recovery in toxin-induced myopathy, shortening regeneration timelines compared to controls [234]. Innovative pre-conditioning approaches, such as near-infrared-mediated NADH priming, further optimized donor mitochondrial bioenergetics and post-transplantation engraftment efficiency in dystrophic muscle [164]. Mechanistically, transplanted mitochondria synergistically augment tissue repair through triad pathways: energetic rescue, redox homeostasis restoration, and immunomodulation via macrophage polarization toward anti-inflammatory phenotypes [163,164,232]. These preclinical findings highlight mitochondrial transplantation as a versatile, cell-free therapeutic paradigm for combatting muscle degenerative pathologies. However, translational validation in large-animal models remains essential to address species-specific regenerative limitations. Together, these cell and cell-derived strategies, centered on translational optimization of both cellular components and delivery systems, have substantially elevated the therapeutic potential of regenerative medicine in skeletal muscle repair, offering strong evidence for future clinical applications.

Several limitations of this review should be acknowledged. First, this is a narrative review, not a systematic one; we did not register a review protocol, conduct a comprehensive quality assessment of primary studies, or follow PRISMA guidelines. The selection of topics and references reflects the authors’ expertise and judgment rather than a quantitative evidence synthesis. Second, the vast majority of mechanistic and therapeutic evidence discussed here is derived from rodent models or in vitro systems. Human data, particularly from randomized controlled trials, remain scarce, and the extent to which preclinical findings translate to patients is uncertain. Third, we have attempted to compare regenerative impairments across multiple disease conditions, but these comparisons are complicated by substantial heterogeneity in study design, injury models, species, and outcome measures across the cited literature. Direct cross-disease comparisons should therefore be interpreted with caution. These limitations notwithstanding, we believe the present review provides a useful integrative framework for understanding shared and disease-specific mechanisms of regeneration failure and for identifying translational priorities.

## 5. Future Perspectives

Integrated strategies that combine gene editing, cell therapy, and biomaterial scaffolds are already being tested preclinically, with early clinical studies starting in some settings. Stratifying patients by disease etiology and molecular subtype could improve outcomes, though the evidence base for personalized approaches is still thin. Engineers, biologists, and clinicians will need to work together more closely if we want to move preclinical findings into real treatments. The big challenges remain: making cell-based therapies last longer, scaling up biomaterial production, and designing trials that can actually detect functional gains in messy, heterogeneous patient populations.

Looking ahead, three areas stand out for skeletal muscle regeneration: AI-driven diagnostics and therapeutic platforms, clinical translation of multifunctional biomaterials, and cross-scale manufacturing. The longer-term vision is a form of personalized therapy that continuously monitors physiology and adapts interventions in real time. New biomaterials are being designed to support mechanical integrity, electrical signaling, and immune modulation all at once. But getting there requires solving some stubborn problems: standardizing biomanufacturing, building robust clinical validation frameworks, and dealing with the ethical side of translational medicine. As collaborative efforts continue to link basic discoveries with clinical use, skeletal muscle regeneration research is gradually bridging the gap between mechanistic discovery and clinical application. Beyond computation, microphysiological systems (MPSs, or “muscle-on-a-chip”) offer a useful platform for pharmacological testing. By mimicking the human regenerative niche, including crosstalk between muscle stem cells, microvasculature, and immune cells, MPSs allow for high-throughput screening of the combination therapies discussed here. This is especially valuable for evaluating long-term safety and synergy in a human-relevant context, though the predictive value of MPSs for clinical outcomes has not been fully established.

On the pharmacology side, a few translational priorities seem most urgent. First, the ongoing experience with GLP-1 receptor agonists illustrates both the promise and the complexity of multi-target strategies: metabolic benefits may come with unintended effects on satellite cell function, and several trials are now testing GLP-1RAs alongside muscle-protective adjuvants to preserve lean mass. Second, senotherapeutics are maturing quickly as complementary approaches that clear senescent cells and restore progenitor activation. Drugs such as navitoclax (ABT-263) or the dasatinib/quercetin combination have shown preclinical efficacy in clearing senescent satellite cells, FAPs, and immune cells, thereby reducing SASP-driven chronic inflammation [80]; rapamycin, used as a senomorphic, offers a preventive strategy by delaying senescence in muscle stem cells. Third, accelerating target validation using multi-omics and single-cell tools, including single-cell RNA-seq, ATAC-seq, and spatial transcriptomics, has already uncovered actionable candidates such as macrophage-derived HGF, the Gli1^+^ satellite cell subpopulation, and the CREG1–AMPKα1 mitophagy axis. Progress will likely require closer coordination between pharmacologists, bioengineers, and clinical trialists, along with trial designs that can detect patient-relevant functional outcomes.

## Figures and Tables

**Table 1:** Summary of Therapeutic Strategies, Molecular Targets, and Regenerative Effects in Skeletal Muscle Repair.

Category	Therapeutic Agent/Strategy	Primary Molecular Target/Pathway	Key Regenerative Effects	Relevant Pathologies	Evidence Stage	References
**Exercise**	Resistance Training	Piezo1; AKT/mTORC1	Enhances protein synthesis and MuSC directional migration	Sarcopenia; Atrophy	Preclinical (animal)	[142,143]
	Endurance Training	miR-1; miR-31	Delays satellite cell senescence; preserves regenerative capacity	Sarcopenia; Aging	Preclinical (animal)	[142,144]
**Bioactive Molecules**	PGE2/15-PGDHi	EP4–CREB axis	Restores aged satellite cell transcriptional networks; enhances muscle mass and strength in elderly	Aging; Sarcopenia	Preclinical (animal)	[83,145]
	GLP-1RAs + 15-PGDHi	PGE2-EP4 signaling axis	Reverses semaglutide-induced MuSC deficit; restores strength	Obesity; Diabetes	Preclinical (mouse)	[146]
	GLP-1RAs + Trevogrumab ± Garetosmab	GDF8/Myostatin and Activin A blockade	Preserves lean mass, enhances fat loss during GLP-1RA-induced weight loss	Obesity	Preclinical (mouse/NHP) + Clinical (Phase 2)	[147,148]
	AICAR (AMPK agonist)	AMPKα1; SIRT1/PGC-1α	Restores mitochondrial homeostasis and autophagic quality	Aging; CKD	Preclinical (animal)	[43,46]
	HGF (Mac_1 derived)	c-Met/Cdkn1b	Drives MuSCs from G0 to GAlert state; triggers activation	Acute Injury	Preclinical (animal)	[57,58]
	Resolvin D1/D2	Macrophage M2 polarization	Modulates inflammatory resolution and fiber type specification	Chronic Inflammation	Preclinical (animal)	[149,150]
**Physical Therapy**	NMES/EMGstim	Myogenic gene expression; ATP release	Enhances myogenic precursor proliferation and fusion; reduces oxidative stress	Aging; Neuropathic injury	Preclinical (in vitro + animal)	[151,152]
	LIPUS	AMPK; Wnt/β-catenin	Promotes M2 polarization and regulates ECM remodeling	Volumetric Muscle Loss	Preclinical (animal)	[153]
	Robotic Mechanotherapy	Neutrophil clearance kinetics	Accelerates transition from inflammatory to myogenic repair phase	Severe/Volumetric Muscle Injury	Preclinical (mouse)	[154]
**Gene Editing**	E-sel/AAV Therapy	E-selectin	Promotes vascular reconstruction and MuSC differentiation	Ischemic Myopathy	Preclinical (animal)	[155]
	miR-204 inhibition/lncMDP1	Pax7; IGF1; CHAC1	Promotes myoblast proliferation, migration, and differentiation	DMD; Atrophy	Preclinical (in vitro + animal)	[156,157]
**Engineered Biomaterials**	Gold Nanoparticles	M2 polarization signaling	Optimizes immunomodulatory niche and restores dystrophic repair	DMD	Preclinical (animal)	[158]
	80Si-BGN (Silicon ions)	Myogenic gene program	Directly stimulates myoblast differentiation via ion release	Volumetric Muscle Loss	Preclinical (in vitro/animal)	[159]
	GHM (MXene-based) Bioink	GelMA/HAMA composite with MXene nanoparticles	Enhances myoblast differentiation and printability; minimal immune rejection	Volumetric Muscle Loss	Preclinical (in vitro + mouse VML)	[160]
	Programmable Immunomodulatory Biomaterials	Spatiotemporal immune signal delivery	Sequentially suppresses pro-inflammatory phase, promotes regenerative transition	Musculoskeletal Injury	Preclinical (animal)	[161]
**Cell and Cell-derived Therapies**	hiPSC-derived EVs	Notch and Wnt signaling	Augments myogenic induction; minimizes immune rejection	DMD; Sarcopenia	Preclinical (in vitro/animal)	[162]
	BMSC-Mito (Mito-transfer)	Oxidative phosphorylation	Energetic rescue; restores redox homeostasis	Denervation; DMD	Preclinical (animal)	[163,164]

## Data Availability

No new data were created or analyzed in this study. Data sharing is not applicable to this article.
